# Encapsulation of *Heracleum persicum* essential oil in chitosan nanoparticles and its application in yogurt

**DOI:** 10.3389/fnut.2023.1130425

**Published:** 2023-06-09

**Authors:** Mojtaba Yousefi, Elham Khanniri, Sara Sohrabvandi, Nasim Khorshidian, Amir M. Mortazavian

**Affiliations:** ^1^Food Safety Research Center (Salt), Semnan University of Medical Sciences, Semnan, Iran; ^2^Department of Food Technology Research, Faculty of Nutrition Sciences and Food Technology, National Nutrition and Food Technology Research Institute, Shahid Beheshti University of Medical Sciences, Tehran, Iran; ^3^Department of Food Science and Technology, Faculty of Nutrition Sciences and Food Technology, National Nutrition and Food Technology Research Institute, Shahid Beheshti University of Medical Sciences, Tehran, Iran

**Keywords:** chitosan nanoparticles, *Heracleum percicum* essential oil, antioxidant activity, antibacterial activity, yogurt

## Abstract

*Heracleum percicum* essential oil (HEO) at various levels was encapsulated in chitosan nanoparticles and its potential application in yogurt was investigated. The values obtained for encapsulation efficiency, loading capacity, mean particle size, and zeta potential of nanoparticles were 39.12–70.22%, 9.14–14.26%, 201.23–336.17 nm, and + 20.19–46.37 mV, respectively. The nanoparticles had spherical shape with some holes as a result of drying process. *In vitro* release studies in acidic solution and phosphate buffer solution indicated an initial burst effect followed by slow release with higher release rate in acidic medium. Results of antibacterial activity revealed that *Staphylococcus aureus* and *Salmonella typhimurium* with inhibition zones of 21.04–38.10 and 9.39–20.56 mm were the most sensitive and resistant bacteria to HEO, respectively. Incorporation of encapsulated HEO into yogurt decreased pH and increased titratable acidity due to stimulation of starters’ activity. Interaction of nanoparticles with proteins decreased syneresis in yogurt. Regarding antioxidant activity, a higher value was observed in yogurt containing encapsulated HEO after 14 days of storage due to degradation and release of essential oil from nanoparticles. In conclusion, application of HEO nanoparticles in yogurt could be a promising approach for development of functional food products such as yogurt with enhanced antioxidant properties.

## Introduction

Yogurt is one of the most popular fermented dairy products and is considered as a healthy food due to high nutritional value and its therapeutic effects (1). Yogurt is a suitable matrix to be fortified with bioactive compounds which can increase its health benefits and consumer acceptance (2). In this respect, various bioactive ingredients including herbal extracts and essential oils have been added.

Essential oils (EOs) are secondary liquid metabolites of plants with lipophilic and volatile nature (3, 4). Recently, due to the consumers demand for replacing synthetic preservatives, EOs have gained attention as natural ingredients in foods with various biological activities, including antimicrobial, antiviral, and antioxidant properties besides flavoring characteristic (5–7).

*Heracleum persicum* commonly known as “Goplar” in Persian has been widely used in traditional medicine to cure cough, fever, insomnia, constipation and skin diseases (8). It has been reported that it has anti-inflammatory, analgesic, antioxidant, and antimicrobial activity (9). Its essential oil contains high amounts of monoterpenes and sesquiterpenes as well as other groups of metabolites such as saponins, glycoside, mucilage, vitamins, and alkaloids that have been from different parts of Golpar (10, 11). However, EOs have low stability and are degraded when exposed to heat, pressure, light and oxygen (12, 13). Encapsulation of EOs can protect them from degradation and interaction with food ingredients, cover their undesirable aroma, and increase their solubility and bioactivity (14).

Chitosan is a non-toxic, biodegradable polymer composed of glucosamine and N-acetyl-glucosamine monomers. In acidic medium, amino groups present in chitosan structure are protonated and can form cross-linkages with negatively charged compounds (15). Reaction of cationic chitosan with anionic sodium tripolyphosphate (TPP) results in formation of nanoparticles (16) that have been widely used in encapsulation of various bioactive compounds such as vitamins (17), eugenol (18), carvacrol (19), *Carum copticum* essential oil (20) and oregano essential oil (21).

In this study, *Heracleum persicum-*loaded particles were fabricated using chitosan and TPP with an electrostatic interaction which is a mild and rapid method with and non-toxic ingredients that make it suitable for biological applications. Also, the positive charge of chitosan nanoparticles enhances the bioavailability of bioactive food ingredients. Moreover, adding *Heracleum persicum* essential oil nanoparticles to yogurt could provide the possibility of the production of a functional food and a new choice for consumers of dairy products. Therefore, this research aimed to develop and characterize nanoparticles with *Heracleum persicum* essential oil as well as investigating its effect on quality characteristics of yogurt.

## Materials and methods

### Materials

Chitosan with medium molecular weight (deacetylation degree 75–85%), sodium tripolyphosphate, Tween 80, and 1-diphenyl-2-picrylhydrazyl (DPPH) were purchased from Sigma-Aldrich (St. Louis, MO, United States). Mueller-Hinton agar (MHA) and Mueller-Hinton broth (MHB) were purchased from Merck (Darmstadt, Germany). *Heracleum percicum* essential oil was provided by Adonis Gol Darou Co. (Tehran, Iran). *Staphylococcus aureus* PTCC 1431, *Bacillus cereus* PTCC 1154, *Escherichia coli* ATCC 1276, and *Salmonella typhimurium* ATCC 14028 were provided by Iranian Research Organization for Science and Technology (Tehran, Iran).

### *Heracleum percicum* essential oil-loaded chitosan nanoparticles

Chitosan nanoparticles loaded with *Heracleum percicum* essential oil were produced according to the method described by Ahmadi et al. (22) with some modifications. 1 g chitosan powder was dissolved in 1% (*v*/*v*) acetic acid solution under magnetic stirring. Tween 80 (1% *v*/*v*) was added to chitosan solution and stirred for 2 h at 40°C to obtain a homogenous mixture. Then, various levels of essential oil (0.25, 0.5, and 0.75 g) were added to chitosan solution and stirred at 400 rpm for 15 min to obtain O/W emulsion with different ratios of chitosan to essential oil of 1:0.5, 1:1, and 1:1.5, respectively. Then, 100 mL TPP solution (1% *w*/*v* in distilled water) was added slowly to the emulsion under magnetic stirring at 500 rpm for 45 min to obtain a cloudy solution. The solution was then centrifuged at 10000 × *g* for 5 min and the obtained nanoparticles were washed with 0.1% Tween 80 solution to remove surface oil and were frozen at −80°C for 2 h and lyophilized with a freeze drier for 14 h (ALPHA 2–4; Christ, Harz, Germany).

### Encapsulation efficiency (EE) and loading capacity (LC)

The level of essential oil in nanoparticles and EE% was determined by UV–Vis spectroscopy (OPTIMA SP-3000 plus, Tokyo, Japan). In order to specify the maximum absorbance of essential oil, wavelength scanning was carried out with a stock solution of *Heracleum percicum* essential oil which indicated a sharp peak at 257 nm. For quantification of essential oil in nanoparticles, a solution containing deionized water containing Tween 80 was added to 15 mg of samples and stirred at 200 rpm at room temperature overnight until complete dissolution. The supernatant was collected after centrifugation at 5000 × *g* for 5 min for determination of absorbance and calculation of essential oil concentration. At the wavelength of maximum HEO absorption, HEO was diluted in ethanol to various concentrations (0.5–5 mg/mL) and the absorbance was measured at each level and calibration curve was drawn. All experiments were performed in triplicate and were reported as mean values. EE and LC were calculated using the following equations:


$$\text{EE}\;\left(\%\right)=\frac{\text{Total amount of loaded essential oil}}{\text{Initial amount of essential oil}\;}\times 100.$$

$$\text{LC}\;\left(\%\right)=\frac{\text{Total amount of loaded essential oil}\;}{\text{Weight of nanoparticles}}\times 100.$$

### Determination of particle size and zeta potential

The measurement of mean particle size of sample was carried out using Malvern Nano ZS (red badge) ZEN 3600 on the basis of dynamic light scattering (DLS). Samples were scattered with double-distilled water (1:100) at a scattering angle 173° and at room temperature. The particle size distribution was determined at 25°C at an angle of 90° and expressed as the polydispersity index (PDI). In order to determine zeta potential, nanoparticles were dispersed in distilled water and measurement was conducted with the aforementioned instrument. All measurement were done in triplicate and the results are presented as mean ± SD.

### Morphological characterization

Scanning electron microscope (SEM; Philips XL30) was used to observe the morphology of nanoparticles. The samples were mounted to the specimen holder with a double-sided adhesive tape and a sputter coater was used to coat the particles with gold. Samples were examined at room temperature at 25 kV.

### *In vitro* HEO release study

The release of HEO from nanoparticles was determined in pH 1.5 (1 mol/L HCL) and pH 7 (phosphate buffer saline). 150 mg of nanoparticles were suspended in 100 mL solution and incubated at 37°C and 150 rpm. At different times, sampling (3 mL) was performed with replacing the volume by fresh solutions. After centrifugation (8000 × g for 8 min), the level of essential oil was measured in the supernatant by a UV–vis spectrophotometer at a wavelength of 257 nm. Cumulative release was calculated during 24 h (23).

### Determination of antibacterial activity

Bacterial stock cultures were activated in Mueller–Hinton Broth (MHB) at 37°C for 24 h. Antibacterial activity was studied by agar well diffusion assay. 100 μL of broth culture containing 10^7^–10^8^ CFU/mL of bacterial cell were inoculated on Mueller–Hinton Agar (MHA) plates. Subsequently, three wells with a diameter of 6 mm were formed on the agar plate and nanoparticles were added to the wells. Plates were incubated at 37°C for 24 h and inhibition-zone diameters (mm) were measured through subtracting the well diameter from the total inhibition zone (24).

### Yogurt preparation

For preparation of stirred-type yogurts, distilled water was added to skim milk powder (1.25% fat) to a total solid content of 13% (*w*/*v*). Then, they were placed in heat treated glass jars at 95°C for 15 min in the water bath. After cooling to the temperature of 42°C, bacterial culture (0.4%, *v*/*v*) containing *Lactobacillus delbrueckii* subsp. *bulgaricus* and *Streptococcus thermophilus* was added and incubated in the water bath at 42°C until pH 4.50 was reached. The freeze-dried nanoparticles with the highest EE% were added at 5% (*w*/*w*) to yogurt formulation. Yogurt samples were stored at 4°C for 21 days.

### Measurement of pH and titratable acidity

The pH of the yogurt samples was determined using a pH meter (Metrohm, Switzerland). For measurement of titratable acidity, 10 g of each yogurt sample was mixed with 75 mL of distilled water and titrated with 0.1 N NaOH using phenolphthalein as indicator. The results were expressed as % of lactic acid.

### Determination of syneresis

For determination of syneresis, 20 g of samples was centrifuged for 10 min at 3000 × g. The syneresis was calculated as the percentage of the volume of the transparent liquid phase (separated from yoghurt) to the weight of the yogurt sample.

### Determination of color

To determinate lightness, a portable colorimeter (ColorFlex EZ, Hunter Lab Reston, United States) was utilized. The L* value was measured according to the Commission Internationale de l’Eclairage (CIELAB system).

### Determination of yogurt antioxidant activity

Yogurt samples (10 g) were diluted by adding 2.5 mL of distilled water (pH was set to 4 by adding 1 M HCl) and stirred. The yogurt was incubated at 45°C for 10 min and then centrifuged at 10,000 rpm for 10 min at 4°C. The supernatant was separated, and the pH was set to 7 by adding NaOH. Centrifugation was done again at 10,000 rpm for 10 min at 4°C, and about 250 μL of the sample was added into 3 mL of the ethanolic solution of DPPH (60 μM) and stirred vigorously. Afterward, it was stored for 5 min at the ambient temperature, and the absorption was measured at 517 nm by spectrophotometer (OPTIMA SP-3000 plus) (24). Antioxidant activity was presented as percentage of DPPH radical scavenging activity and calculated using the following formula:


$$\text{DPPH radical scavenging activity}\%=\;\;\left[{\text{A}}_{\text{control}}-\;{\text{A}}_{\text{ sample}}/{\text{A}}_{\text{control}}\right]\times 100.$$

### Statistical analysis

Difference between mean values was analyzed using ANOVA and Duncan multiple range tests. The SPSS software (ver. 22.0, SPSS Inc., United States) was used to analyze the experimental data. Differences at *p <* 0.05 were considered to be significant.

## Results and discussion

### Encapsulation efficiency and loading capacity of HEO-loaded nanoparticles

The levels of EE and LC in different nanoparticles are illustrated in Table 1. Based on the UV–vis spectrophotometry results, the level of *Heracleum percicum* essential oil influenced encapsulation efficiency. EE% of 39.12–70.22% was obtained and increasing the level of essential oil caused a decrease of EE% due to the limited entrapping capacity and saturation of particles with essential oil. Similar results have been reported in previous studies entrapping *Carum copticum* essential oil (20), cinnamon essential oil (25), bitter orange oil (26), cumin (27) and nettle essential oil (28). Hadidi et al. (29) reported the highest EE% of clove essential oil at 1:0.5 ratio of chitosan/essential oil. Yilmaz et al. (30) used electrospraying technique to prepare chitosan nanoparticles containing *Origanum vulgare* essential oil and reported EE% of 70–79.6% that was higher compared to other methods such as desolvation, ionic gelification, and gelation.

**Table 1:** Encapsulation efficiency, loading capacity, mean particle size, zeta potential, and polydispersity index (PDI) of loaded and unloaded chitosan nanoparticles.

CS:HEO ratio	EE%	LC%	Mean particle size (nm)	Zeta potential (mV)	PDI
1:0.00	0.00	0.00	201.23 ± 23.41	+ 46.37 ± 4.65	0.441 ± 0.005
1:0.50	70.22 ± 2.31	9.14 ± 0.51	254.35 ± 20.19	+ 36.29 ± 3.91	0.415 ± 0.021
1:1.00	58.19 ± 1.73	11.38 ± 1.84	289.10 ± 27.61	+ 29.15 ± 5.11	0.367 ± 0.001
1:1.5	39.12 ± 2.65	14.26 ± 2.57	336.17 ± 30.29	+ 20.19 ± 2.40	0.318 ± 0.003

Unlike EE, LC percentage increased significantly (*p* < 0.05) from 9.14 to 14.26% by increasing HEO initial concentration. This result confirms the results obtained in encapsulation of carvacrol (19), clove essential oil (31) and cumin essential oil (27).

### Particle size and zeta potential

**Figure 1:**
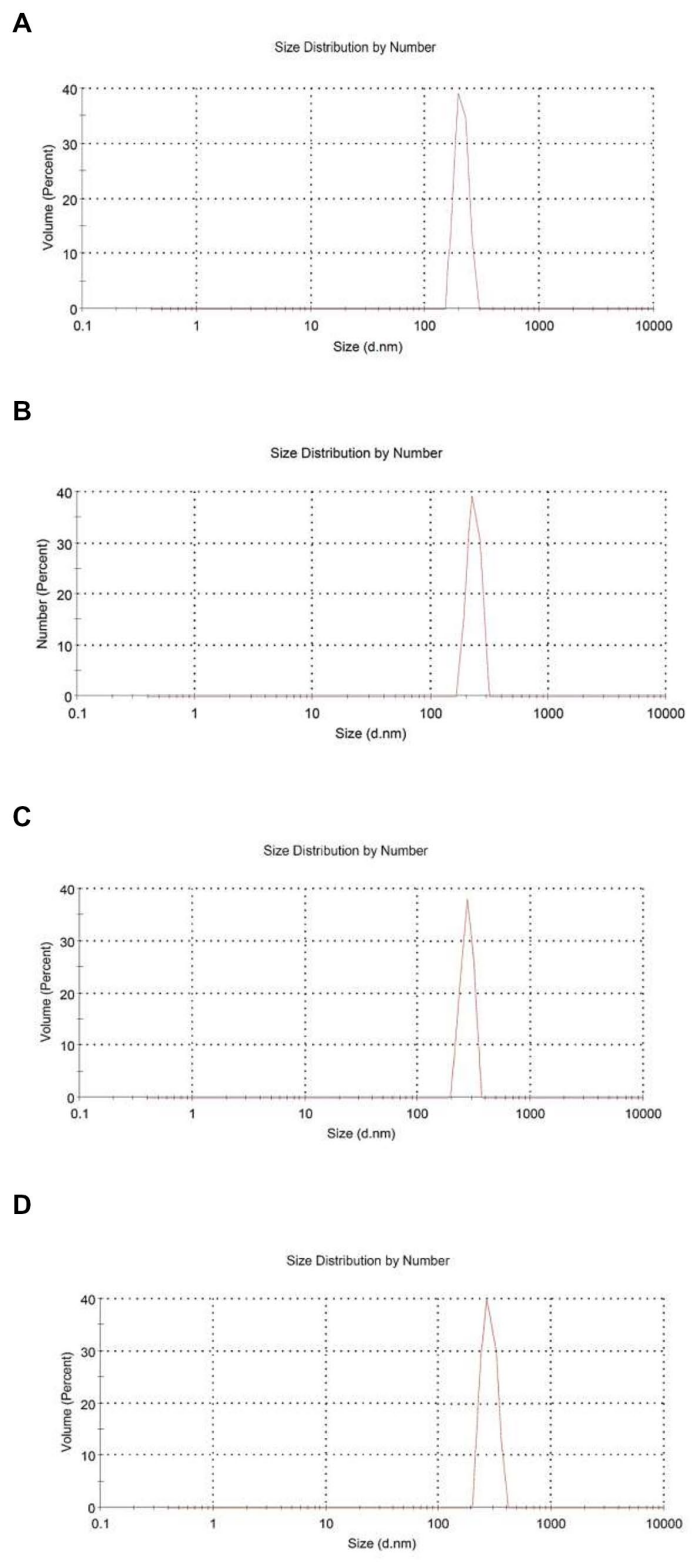
Size distribution by number for chitosan nanoparticles with different chitosan: *Heracleum percicum* essential oil (HEO) ratios, blank chitosan nanoparticles **(A)**, 1:0.5 **(B)**, 1:1 **(C)**, and 1:1.5 **(D)**.

From Table 1, the mean particle size of HEO-loaded particles was in the range of 201.23–336.17 nm and had a narrow size distribution (Figure 1). According to Table 1, increasing the level of essential oil enhanced the particle size. Similar results have been reported in encapsulation of summer savory essential (32), garlic essential oil (33), *Arrabidaea chica* extract (34), *Cinnamomum zeylanicum* (35) and tarragon essential oils (36). In a study by Soltanzadeh et al. (37), entrapment of pomegranate peel extract in chitosan nanoparticles resulted in larger particle size due to the possible diffusion of extract to the surface of nanoparticles that caused sticking and aggregation. Moreover, it has been reported that chitosan molecular weight and concentration were the factors influencing the particle size. At high levels of chitosan, many chitosan molecules participate in cross-linking and the intermolecular hydrogen bond attraction is stronger, and the electrostatic repulsion is insufficient, leading to the formation of larger particles upon the addition of TPP (38).

Zeta potential is indicative of surface charge of nanoparticles and their stability. Values higher than 30 mV causes repulsion between particles and increase the stability (39). According to Table 1, nanoparticles without essential oil had a zeta potential of + 43.57 mV due to the presence of NH_3_^+^ groups of chitosan which declined in HEO-loaded nanoparticles (+ 20.19 mV). It has been reported that adsorption of essential oil on the particle surfaces masked the free amine groups of chitosan and decreased zeta potential (40). Similar results have been reported in encapsulation of pomegranate peel extract (37), thyme essential oil (41), and lime essential oil and *Schinus molle* L. essential oil (42, 43). López-Meneses et al. (42) found zeta potential of + 40.2 mV in *Schinus molle* essential oil-loaded nanoparticles. Hesami et al. (44) reported zeta potential in the range of + 26.46 to + 33.1 mV in chitosan nanoparticles containing *Chelidonium majus* EO.

The polydispersity index (PDI) was determined to assess the particle size distribution in suspensions. A lower PDI is indicative of more homogenous particle size distribution and diameter uniformity. Our data showed that PDI of nanoparticles was in the range of 0.318–0.441 which indicated the homogeneity of the samples and uniform particle size distribution.

### Morphology of nanoparticles

**Figure 2:**
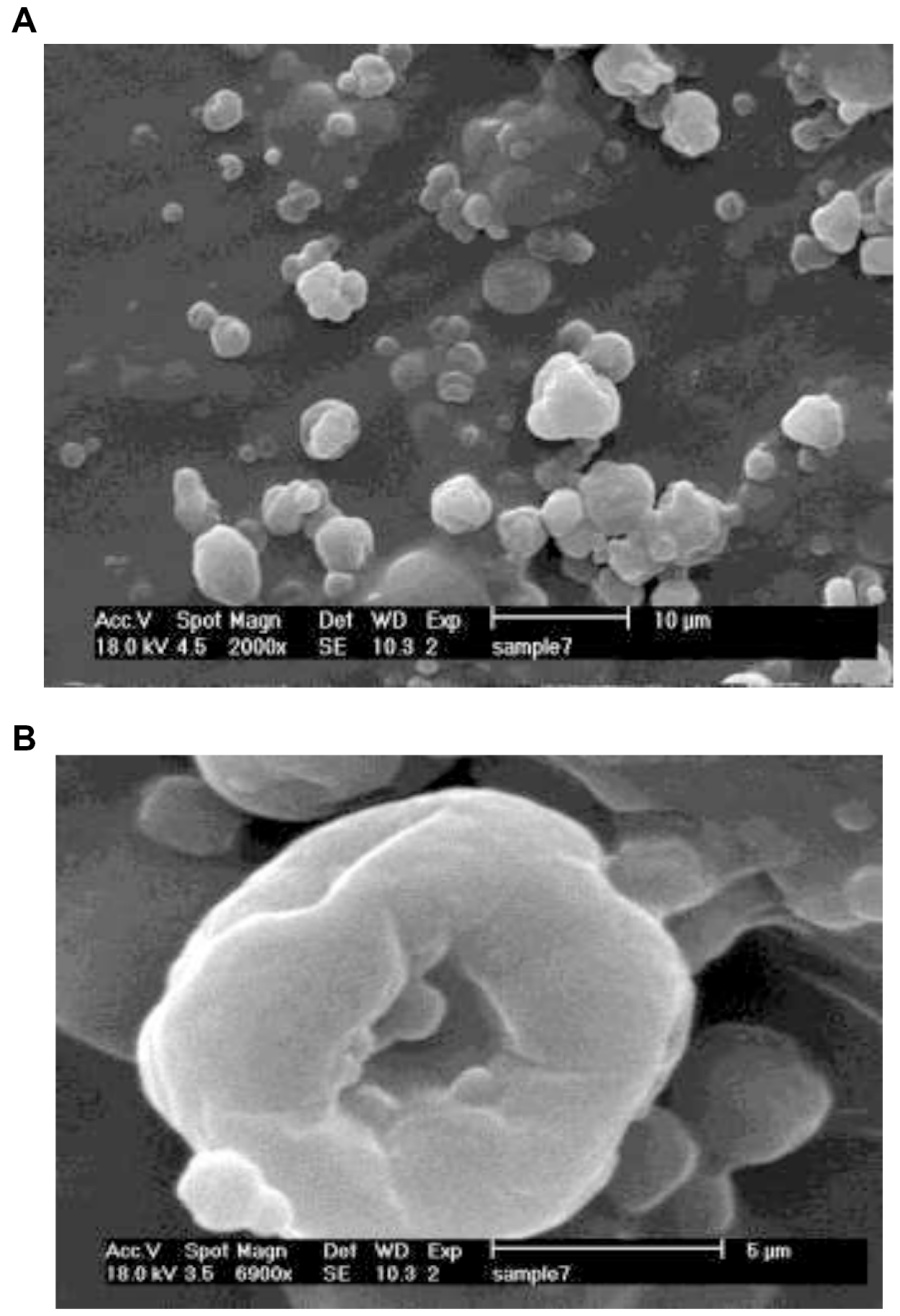
Scanning electron microscope (SEM) images of HEO-loaded chitosan nanoparticles; 2000 × **(A)** and 6900 × **(B)**.

Figure 2 demonstrates the morphology of essential oil-loaded nanoparticles. The particles had spherical shape and in some cases with holes that have been formed as a consequence of drying process (45). This is in line with the findings obtained by Ahmadi et al. (22) and Saikia et al. (46).

### Release of HEO from chitosan nanoparticles

**Figure 3:**
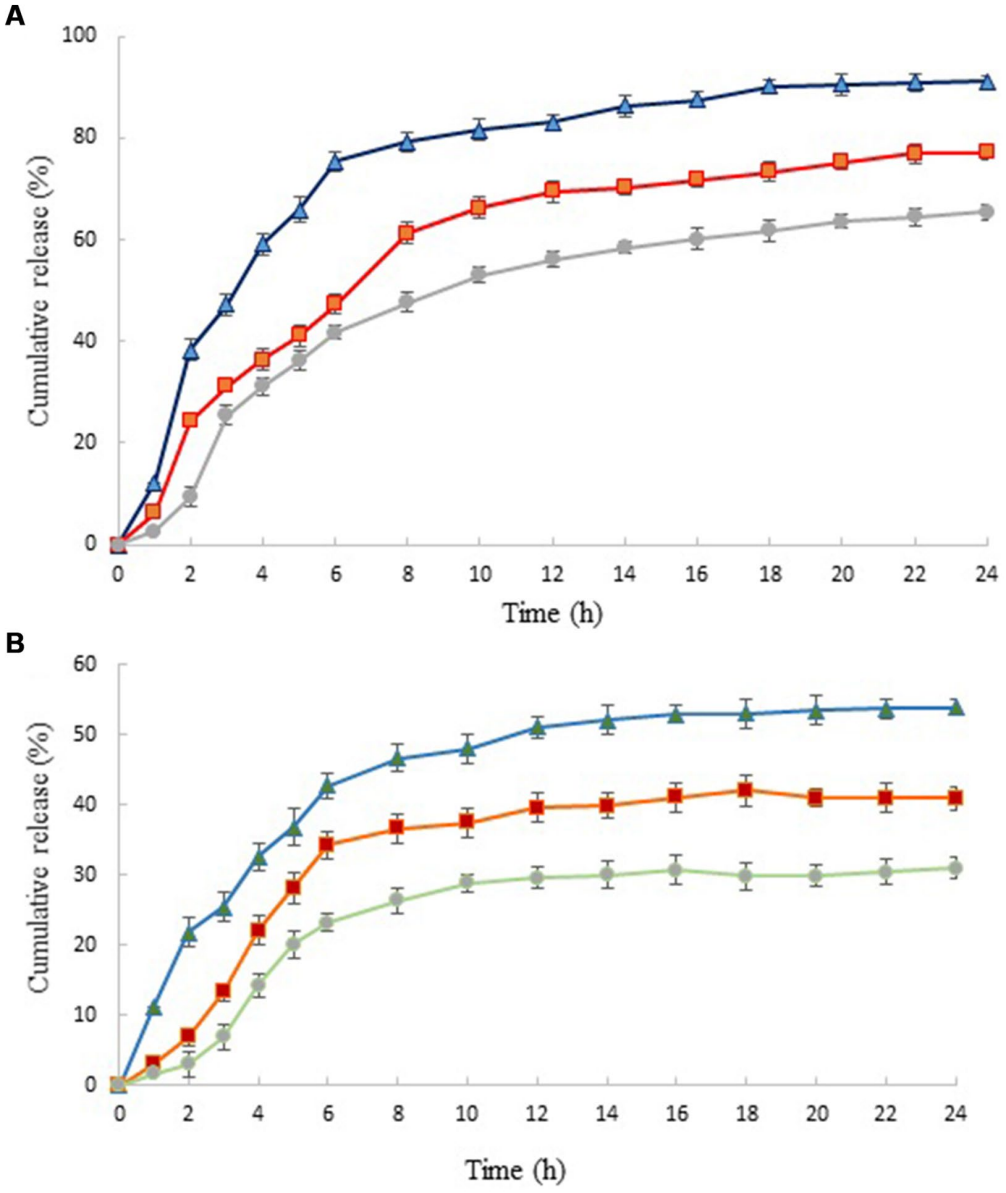
*In vitro* release profiles of HEO from chitosan nanoparticles prepared using different weight ratio of chitosan to ZEO: 1:0.5 (▲), 1:1 (■) and 1:1.5 (●) in HCl **(A)** and phosphate buffer saline **(B)**. Values were expressed as mean ± standard deviation.

The release of HEO from chitosan nanoparticles at different pHs is shown in Figure 3. The release profile showed a two-step biphasic process, including an initial burst release followed by a slow release. The initial burst was due to the HEO molecules adsorbed on the nanoparticles’ surface and the ones located near the surface (47). Several studies have suggested that polymer degradation is the primary mechanism by which chitosan nanoparticles release essential oils into the external environment (48). According to Pereira et al. (49), the slow-release phase was the consequence of the diffusion of encapsulated essential oil molecules from the nanoparticle core to the dissolution medium through interconnected pores and channels in the polymer matrix that along with hydrophobic nature of essential oil, showed a slower water absorption rate and decreased polymer degradation.

Similar results have been reported by Shetta et al. and Mehran et al. (50, 51). It has been reported that this phenomenon was due to the concentration difference of essential oil in the first and second stages of the release test (52). The release profile of nanoparticles revealed that HEO released faster from nanoparticles with lower level of essential oil. In acidic solution (pH 1.5) and in nanoparticles with the lowest level of HEO (chitosan: HEO 1:0.5), burst release occurred within 2 h and 91.13% of essential oil released from nanoparticles after 24 h. Increasing the HEO level caused a decrease in cumulative release from 91.13 to 65.38%. This can be attributed to the effect of particle size on the release rate. Greater surface-to-volume ratio in small nanoparticles resulted in faster release of essential oil adsorbed on the surface (21). After this stage, the release of HEO reached a plateau that was due to the diffusion of HEO dispersed in the chitosan nanoparticles (53). The same trend was observed in the release of HEO from nanoparticles in phosphate buffer solution. A burst release of 21.78% after 2 h was obtained in nanoparticles containing the lowest level of HEO that reached to 53.98% after 24 h. Our findings revealed that the release of HEO was higher in acidic solution compared to neutral medium. It has been declared that increasing the concentration of hydrogen ions in low pH led to increase of repulsive force between protonated free amino groups (NH_3_^+^), weakening the chitosan structure and consequently, higher release of essential oil (52).

Similarly, Karimirad et al. (54) reported faster release of cumin essential oil from chitosan nanoparticles in the acetate buffer compared to phosphate buffer solution that was attributed to swelling and partial dissolution of polymers as well as an increase in particle surface.

### Antibacterial activity

Antimicrobial activity of HEO-loaded nanoparticles against selected bacteria is shown in Table 2. HEO in free form inhibited all bacteria and the antibacterial activity increased by increasing the essential oil concentration. According to (8), *Heracleum persicum* essential oil possesses antibacterial activity against Gram-positive and Gram-negative bacteria. Essential oils exert their inhibitory activity through changing cell permeability, disrupting the enzymes responsible for energy production, and interrupting the protein motive force that finally results in the cell death (55). The major compounds in *Heracleum persicum* essential oil which exert antimicrobial activity include sesquiterpene (e.g., caryophyllene oxide), monoterpene hydrocarbons (e.g., p-cymene, γ-terpene, αand β-pinene, and limonene, etc.), and oxygenated monoterpenes (e.g., iso-bornyl acetate, linalool, n-octanol, and terpinene-1-ol-4, etc.) in their volatile fraction (56). Higher antibacterial activity was observed against *S. aureus*, *B. cereus*, *E. coli*, and *S. typhimurium* with mean inhibition zone of 21.04–38.10, 19.51–31.44, 14.46–26.17, and 9.9–20.56 mm, respectively, by essential oil-loaded nanoparticles compared to free essential oil. These findings are as a result of controlled release of HEO from nanoparticles besides the presence of positive-charged groups in chitosan which bind to the anionic groups on the cell surface of bacteria (20, 57). In agreement with these results, Granata et al. (41) reported higher antibacterial activity of chitosan nanoparticles containing thyme and oregano essential oil compared to pure essential oils. It was declared that antibacterial activity of essential oil-loaded chitosan nanoparticles was dependent on several factors, including composition of essential oils, diffusion, surface charge, and size which do not always act in a synergistic way.

**Table 2:** Antibacterial activity of free HEO, unloaded and HEO-loaded chitosan nanoparticles.^a^

Samples	Inhibition zone (mm)			
	*Staphylococcus aureus*	*Bacillus cereus*	*Escherichia coli*	*Salmonella typhimurium*
HEO-0.25	17.23 ± 1.11^f^	14.10 ± 1.01^f^	12.16 ± 1.70^f^	9.61 ± 0.88^f^
HEO-0.50	23.18 ± 1.54^d^	22.36 ± 1.38^d^	19.13 ± 1.21^d^	15.14 ± 1.05^d^
HEO-0.75	32.11 ± 1.07^b^	27.71 ± 1.19^b^	22.67 ± 1.37^b^	18.15 ± 1.07^c^
Blank chitosan nanoparticles	7.22 ± 0.08^g^	8.73 ± 0.99^g^	9.34 ± 1.10^g^	9.11 ± 0.98^f^
HEO-loaded chitosan nanoparticles-1: 0.5	21.04 ± 1.43^e^	19.51 ± 1.28^e^	14.46 ± 1.08^e^	9.39 ± 0.99^e^
HEO-loaded chitosan nanoparticles-1: 1	26.13 ± 1.67^c^	25.36 ± 2.17^c^	20.76 ± 2.01^c^	19.23 ± 1.10^b^
HEO-loaded chitosan nanoparticles-1: 1.5	38.10 ± 1.12^a^	31.44 ± 2.02^a^	26.17 ± 1.30^a^	20.56 ± 2.16^a^

a Mean of three replications ± standard deviation.

Values within each column with different letters are significantly different (*p* < 0.05).

Encapsulated *Carum copticum* essential oil in chitosan nanoparticles demonstrated higher antibacterial activity with average inhibition zone of 11.3, 12.3, 10.7, 10, 9.5, and 8 mm against *S. aureus*, *Staphylococcus epidermidis*, *Bacillus cereus*, *E. coli*, *S. typhimurium*, and *Proteus vulgaris*, respectively, compared to the free essential oil (20).

As is evident in Table 2, blank chitosan nanoparticles showed antibacterial effect against the tested bacteria. Moreover, it is evident that with enhancing HEO amount, the inhibition zone was augmented significantly (*p* < 0.05) and nanoparticles containing the highest level of HEO, indicated the greatest inhibition zone for all bacteria. The highest and the lowest resistance to HEO-loaded nanoparticles were recorded for *S. typhimurium* and *S. aureus*, respectively.

Regarding synthetic preservatives, Oladapo et al. (58) reported an inhibition zone of 21 and 12 mm for *S. aureus* and *E. coli* by application of potassium sorbate. In another report by Ekhtelat et al. (59), inhibition zone of 13 mm was observed for *S. aureus* by using sodium benzoate. Citric acid solution created inhibition zones of 2.07 and 4.27 mm for *S. aureus* and *E. coli*, respectively (60). These results supported previous reports about more susceptibility of Gram-positive bacteria compared to Gram-negative ones which can be attributed to the thin membrane in Gram-negative bacteria composed of lipopolysaccharides and a thin peptidoglycan layer (35, 61).

### pH and titratable acidity of yogurt samples

A change in pH and acidity of yogurt samples during storage is demonstrated in Table 3. A decreasing trend in pH and an increase in acidity were observed in yogurt samples during storage. Incorporation of free essential oil or nanoparticles had no significant effect on pH and acidity of samples.

**Table 3:** Changes in pH, acidity, and syneresis of yogurt samples during storage.

Sample	pH				Acidity (% lactic acid)				Syneresis (%)			
	1	7	14	21	1	7	14	21	1	7	14	21
Control	4.50 ± 0.03^aD^	4.38 ± 0.01^aC^	4.21 ± 0.02^aB^	4.10 ± 0.01^aA^	1.02 ± 0.01^aA^	1.06 ± 0.00^aB^	1.15 ± 0.02^aC^	1.26 ± 0.01^aD^	13.23 ± 1.25^aA^	15.89 ± 0.96^aB^	25.12 ± 1.16^bC^	29.19 ± 1.04^bD^
Yogurt with free HEO	4.51 ± 0.01^aD^	4.47 ± 0.02^bC^	4.42 ± 0.02^bB^	4.39 ± 0.01^cA^	1.03 ± 0.02^aA^	1.09 ± 0.02^abB^	1.20 ± 0.03^bC^	1.18 ± 0.02^bC^	13.34 ± 1.56^aA^	14.69 ± 1.78^aA^	23.10 ± 0.87^bB^	28.25 ± 1.11^bC^
Yogurt with encapsulated HEO	4.52 ± 0.02^aC^	4.45 ± 0.01^bC^	4.41 ± 0.00^bA^	4.34 ± 0.01^bA^	1.10 ± 0.03^bA^	1.12 ± 0.03^bA^	1.29 ± 0.01^cB^	1.36 ± 0.03^cC^	12.89 ± 0.98^aA^	13.47 ± 1.45^aA^	16.27 ± 1.45^aB^	20.72 ± 1.21^aC^

According to (62), changes of pH evaluated during 21 days of storage showed a decrease from 4.5 to 4 at the end of storage due to the activity of lactic acid bacteria. In a study by Maleki et al. (63), decrease of pH values and increase of acidity in yogurt samples with and without encapsulated *Tragopogon Collins* extract with increasing time has been reported. It was implied that metabolic activity of yogurt bacteria increased due to the presence of extract. Similar results have been obtained by Karimi Sani (64) who reported a decrease in pH of yogurt samples containing encapsulated *Melissa officinalis* essential oil during storage. It was stated that release of bioactive compounds from nanoparticles or free essential oil could stimulate the activity of starter cultures. These findings are in accordance with results obtained by Ghorbanzade et al. (65–67).

### Syneresis of yogurt samples

Syneresis is considered as one of the main defects in yogurt that leads to separation of serum or whey and shrinkage of the three-dimensional structure of the protein network. As shown in Table 3, syneresis increased in all treatments over time that is related to the rearrangement of casein leading to increasing particle connections, which results in network compress and shrinkage as well as serum separation. The highest syneresis value was obtained for control yogurts while samples containing free essential oil and encapsulated essential oil showed the lowest syneresis. In a study by Karimi Sani (64), an increase in syneresis rate was reported in yogurt samples containing encapsulated *Melissa officinalis* essential oil which decreased on the final days of storage. This phenomenon was explained by the interactions of nanoparticle wall material and proteins. The increase in whey protein to casein ratio could induce the shrinkage of the gel, subsequently augmenting whey separation. Also, it was announced that starter cultures could hydrolyze the proteins resulting in disappearing the internal bonds of casein and enhancing syneresis. Similar results have been reported by Akgün et al. (62) and de Moura et al. (68).

### Color of yogurt samples

Lightness (L*) of control yogurt, yogurt with free essential oil, and encapsulated essential oil are illustrated in Table 4. The highest and the lowest L* values were observed in control samples and yogurt containing free HEO on day 1 and 21, respectively. Addition of free and encapsulated HEO to yogurt decreased lightness which continued during storage. Accordingly (67) reported a decrease in L* of control yogurt samples and containing encapsulated beetroot extract. Tavakoli et al. (66) reported the highest lightness value in control yogurt and a decreasing trend was observed during storage. It was highlighted that color indices were correlated with pH and L* decreased by reduction of pH. In another study by de Campo et al. (69), a decrease was observed in yogurt containing zeaxanthin nanoparticles on day 7 with stable value until day 21, followed by an increase on day 28. The decrease of L* values was attributed to the casein in milk. The interaction of protein with bacterial proteases and proteolysis led to decrease of L* during storage.

**Table 4:** Changes in L^*^ of yogurt during storage.

Lightness	Control sample	Yogurt with free HEO	Yogurt with encapsulated HEO
Day 1	91.86 ± 0.7^cC^	73.15 ± 1.54^bcA^	80.24 ± 1.06^bB^
Day 7	88.53 ± 2.14^bC^	70.36 ± 2.43^bA^	79.31 ± 2.15^bB^
Day 14	83.17 ± 1.56^aC^	68.18 ± 1.93^abA^	77.39 ± 1.13^abB^
Day 21	80.49 ± 2.11^aC^	66.43 ± 0.98^aA^	75.27 ± 1.46^aB^

### Antioxidant activity of yogurt samples

Antioxidant activity of yogurt samples is shown in Table 5. The results indicated that antioxidant activity decreased in control and samples containing free HEO during storage. In addition, yogurt containing free and encapsulated HEO showed higher antioxidant activity than control samples. Antioxidant properties of control yogurt can be ascribed to the presence of catalase and super oxidase enzymes, casein, and lactic acid bacteria (70). The antioxidant activity of samples containing HEO was due to the presence of anethole and myristicin (71). Also, it was revealed that antioxidant activity of yogurt with free HEO was higher than samples containing encapsulated HEO till day 7 of storage, but after that, antioxidant activity of yogurt with encapsulated HEO was a bit higher than samples containing free HEO. A decreasing trend in antioxidant activity of yogurt with free HEO was a result of destruction of bioactive phenolic compounds during storage as well as fermentation effect of starter bacteria. After 14 days of storage, antioxidant activity of yogurt containing encapsulated HEO increased due to the degradation and release of bioactive compounds from nanoparticles. These findings are in accordance with the results obtained by Maleki et al. (63), Tavakoli et al. (66), and Flores-Mancha et al. (67).

**Table 5:** Antioxidant activity of yogurt samples during storage.

Antioxidant activity	Control sample	Yogurt with free HEO	Yogurt with encapsulated HEO
Day 1	22.16 ± 0.98^dA^	57.25 ± 1.08^dB^	50.34 ± 1.06^dC^
Day 7	18.55 ± 1.04^cA^	52.69 ± 1.13^cB^	52.31 ± 1.28^cB^
Day 14	15.87 ± 1.16^bA^	47.61 ± 0.98^bB^	56.44 ± 1.02^bC^
Day 21	13.69 ± 1.11^aA^	43.13 ± 1.22^aB^	61.27 ± 1.18^aC^

## Conclusion

In the present study, chitosan nanoparticles containing HEO were fabricated with spherical shape and EE% of 39.12–70.22%, loading capacity of 9.14–14.26%, and particle size of 201.23–336.17 nm. Increasing the level of essential oil decreased EE% and increased LC%. Increasing HEO to chitosan mass ratio resulted in an increased particle size and decreased zeta potential. Release studies demonstrated an initial sudden release followed by a sustained HEO release and the effect of HEO concentration and pH on release rate. The release of HEO from nanoparticles was faster at lower level of essential oil due to greater surface-to-volume ratio in small nanoparticles. It was demonstrated that the release of HEO was higher in acidic solution compared to neutral medium. HEO in encapsulated form showed greater antibacterial activity against Gram-positive and Gram-negative bacteria due to controlled release of HEO from nanoparticles in addition to the antibacterial activity of chitosan. The highest and the lowest resistance to HEO-loaded nanoparticles were recorded for *S. typhimurium* and *S. aureus*, respectively. Addition of encapsulated HEO to yogurt increased activity of starter bacteria which resulted in decrease of pH and increase of acidity during storage. Also, the syneresis in yogurt containing encapsulated HEO was lower as a consequence of the interactions between nanoparticles wall material and proteins. Addition of free and encapsulated HEO to yogurt decreased lightness which continued during storage. Also, encapsulated HEO led to a higher antioxidant activity in yogurt samples due to the release of bioactive phenolic compounds from nanoparticles in yogurt. To conclude, *Heracleum persicum* essential oil can be entrapped in chitosan nanoparticles with acceptable encapsulation efficiency for utilization in food products such as yogurt to enhance their functional healthy properties and consumer acceptance. Also, the prepared chitosan nanoparticles loaded with *Heracleum percicum* essential oil could be a plant-based alternative to synthetic preservatives for control of common food pathogens. These nanoparticles can mask the unpleasant taste, increase the stability of essential oils along with controlled release in food products. However, future investigations are required to assess the stability of incorporated nanoparticles in various food products as well as their effect on quality and sensory characteristics of final product.

## Data availability statement

The original contributions presented in the study are included in the article/supplementary material, further inquiries can be directed to the corresponding authors.

## Author contributions

NK and AM designed the study. MY, EK, and NK carried out the experiments and collected the data. SS, MY, EK, and NK wrote the original draft. AM revised the manuscript. All authors listed have approved the manuscript for publication, contributed to this article, and approved the submitted version.

## Funding

Research reported in this publication was supported by Elite Researcher Grant Committee under award number 987781 from the National Institutes for Medical Research Development (NIMAD), Tehran, Iran.

## Conflict of interest

The authors declare that the research was conducted in the absence of any commercial or financial relationships that could be construed as a potential conflict of interest.

## Publisher’s note

All claims expressed in this article are solely those of the authors and do not necessarily represent those of their affiliated organizations, or those of the publisher, the editors and the reviewers. Any product that may be evaluated in this article, or claim that may be made by its manufacturer, is not guaranteed or endorsed by the publisher.
